# The Influence of Pathological Mutations and Proline Substitutions in TDP-43 Glycine-Rich Peptides on Its Amyloid Properties and Cellular Toxicity

**DOI:** 10.1371/journal.pone.0103644

**Published:** 2014-08-04

**Authors:** Chia-Sui Sun, Cindy Yu-Hsiang Wang, Bryan Po-Wen Chen, Ruei-Yu He, Gerard Chun-Hao Liu, Chih-Hsien Wang, Wenlung Chen, Yijuang Chern, Joseph Jen-Tse Huang

**Affiliations:** 1 Taiwan International Graduate Program in Molecular Medicine, National Yang-Ming University and Academia Sinica, Taipei, Taiwan; 2 Institute of Chemistry, Academia Sinica, Taipei, Taiwan; 3 Institute of Biomedical Sciences, Academia Sinica, Taipei, Taiwan; 4 Institute of Biochemistry and Molecular Biology, National Yang-Ming University, Taipei, Taiwan; 5 Department of Chemistry, National Taiwan University, Taipei, Taiwan; 6 Department of Applied Chemistry, National Chiayi University, Chiayi, Taiwan; Consejo Superior de Investigaciones Cientificas, Spain

## Abstract

TAR DNA-binding protein (TDP-43) was identified as the major ubiquitinated component deposited in the inclusion bodies in amyotrophic lateral sclerosis (ALS) and frontotemporal lobar degeneration with ubiquitin-positive inclusions (FTLD-U) in 2006. Later on, numerous ALS-related mutations were found in either the glycine or glutamine/asparagine-rich region on the TDP-43 C-terminus, which hinted on the importance of mutations on the disease pathogenesis. However, how the structural conversion was influenced by the mutations and the biological significance of these peptides remains unclear. In this work, various peptides bearing pathogenic or *de novo* designed mutations were synthesized and displayed their ability to form twisted amyloid fibers, cause liposome leakage, and mediate cellular toxicity as confirmed by transmission electron microscopy (TEM), circular dichroism (CD), Thioflavin T (ThT) assay, Raman spectroscopy, calcein leakage assay, and cell viability assay. We have also shown that replacing glycines with prolines, known to obstruct β-sheet formation, at the different positions in these peptides may influence the amyloidogenesis process and neurotoxicity. In these cases, GGG308PPP mutant was not able to form beta-amyloid, cause liposome leakage, nor jeopardized cell survival, which hinted on the importance of the glycines (308–310) during amyloidogenesis.

## Introduction

TDP-43 (TAR DNA-binding protein) is the major pathological protein in amyotrophic lateral sclerosis (ALS) and frontotemporal lobar degeneration with ubiquitin-positive inclusions (FTLD-U) [1], [2]. Human TDP-43 is a DNA/RNA binding protein which includes two RNA recognition motifs (RRMs), N-terminus, and a C-terminus containing both glycine-rich and glutamine/asparagine (Q/N)-rich regions. Recently, TDP-43 has been reported to play multiple roles in the cellular system such as mRNA splicing, stabilization, degradation, and transportation [3]–[7]. The histopathological and biochemical signatures of TDP-43 in ALS patients include hyper-phosphorylation, ubiquitinations, and accumulation of cytoplasmic inclusions accompanied with TDP-43 C-terminus fragments in the neurons and/or glial cells of the affected region [1]. Besides ALS and FLTD-U, TDP-43 proteinaceous inclusions have been found as a secondary pathological feature in other diseases including Alzheimer's disease, Huntington's disease, Parkinson's disease, and hippocampal sclerosis, suggesting the broad impact of TDP-43 proteinopathy on the neurodegenerative disorders [8]–[10].

More than 40 pathological TDP-43 mutations were found in the C-terminal domain, which indicated the intrinsic disordered propensity of this region in TDP-43 proteinopathy [11]. Some of these mutations, such as Q331K, M337V, Q343R, N345K, R361S, and N390D, have been proven to correlate directly with either the cytotoxicity or the formation of inclusions in different experiments [12]–[16]. Recently, a breakthrough in delineating the biological impact of the pathological mutation A315T in ALS patients has been characterized in detail. This mutant is able to promote protein aggregation, amyloid fibrillation, locomotive dysfunction, and motor neuron death combined with axonal damage, revealing the possible amyloidogenic and neurotoxic properties of TDP-43 mutants in ALS pathogenesis [17], [18]. Though many mutations have been identified in this protein, their specific roles in TDP-43 proteinopathy as well as their biological significance remain vague.

In this study, we try to characterize the structure, amyloid properties, membrane permeabilization ability, and biological properties of clinically-related TDP-43 mutants including G294V (familial mutation) and G295S (sporadic mutation). Since it has been shown that the addition of proline residues may block β-sheet propensity and prevent amyloidogenesis, various glycines were replaced by prolines in TDP-43 glycine-rich peptides (G294P, GGG294PPP, and GGG308PPP) to confirm the impact of proline substitutions in the peptide aggregation and cytotoxicity. While GGG294PPP was selected due to the frequent pathological mutations in residues 294 and 295, GGG308PPP was chosen for the amyloidogenic property in the specific region (residue 307–322) of TDP-43 [19]. Our result indicated the impact of pathological and *de novo* designed mutations in the amyloid formation and shed light on the possible peptide design in suppressing TDP-43 proteinopathy in the future.

## Materials and Methods

### Peptide preparation and identification

All peptides (D1, G294A, G294V, G295S, G294P, GGG294PPP, GGG308PPP) were synthesized by the batch FMOC polyamide method on a peptide synthesizer (PS3). Rink amide AM resin was selected as the solid support. After cleaved from resin, crude peptides were purified by high-performance liquid chromatography (HPLC). Peptide purity was confirmed by HPLC and Matrix-Assisted Laser Desorption/Ionization (MALDI) mass spectroscopy (> 95% purity). FITC (Fluorescein isothiocyanate)-Ahx(Aminohexanoic) attached G295S and FITC (Fluorescein isothiocyanate)-Ahx(Aminohexanoic) attached GGG308PPP were provided by the peptide center (Institute of Biochemistry, Academic Sinica).

### Fibril formation

Peptide solutions (D1, G294A, G294V, G295S, G294P, GGG294PPP, and GGG308PPP) (50 µM) were dissolved in phosphate buffer (70 mM KCl, 20 mM sodium phosphate, pH 7.0) and determined their concentration by UV spectrometry. All peptides were incubated at 37°C for the indicated times.

### Electron microscopy

After incubation at 37°C for 2 weeks, 5 µL of each fibril solutions (D1, G294V, G295S, G294P, GGG294PPP, and GGG308PPP) were absorbed onto 300 mesh glow-charged, formvar- and carbon-coated copper grids for 4 minutes. The sample was then negatively stained with 2% (w/v) uranyl acetate for 1 min. After drying, all samples were examined under Hitachi H-7000 electron microscope at 75 kV.

### Circular Dichroism (CD) spectroscopy

50 µM of peptides were dissolved in phosphate buffer and incubated at 37°C. 200 µL of TDP-43 peptide samples were collected in the 1-mm pathlength quartz cuvette and measured between 200 to 260 nm by J-815 CD spectrometer (JASCO, Japan). CD measurements were carried out with a speed of 100 nm/min at continuous scanning mode and averaged for three runs.

### FT-Raman measurement

Fourier transform-Raman (FT-Raman) spectra of TDP-43 C-terminal fragment fibril samples were obtained by Bruker RFS-100 FT-spectrophotometer (Bruker Optik GmbH, Lubeck, Germany). Diode laser pump (Nd: YAG laser) was used to generate continuous-wave near-infrared excitation (1064 nm). Raman spectra were recorded with 100 mW of laser power. 500 interferograms were co-added at a resolution of 4 cm^−1^ with a sampling period for about 15 minutes. The spectra in the 1520–1720 cm^−1^ region were subjected to numerical curve fitting (Grams/386; Galactic Ind. Co.). The band shapes were approximated by a Lorentz function and the baseline was approximated to a straight line between two points at 1520 and 1720 cm^−1^, chosen at both sides of the band envelope. The Raman intensity ratio was calculated by the average of at least three measurements. FT-Raman spectra reported in this study were based on the raw spectra without smoothing, normalization, or baseline correction.

### Thioflavin T (ThT) binding assay

The formation of amyloid fiber was monitored by ThT binding assay. 50 µM of peptides were incubated at 37°C for 7 days. At day 7, 2 mM Thioflavin-T (ThT) stock solution was prepared in 140 mM KCl, 100 mM sodium phosphate buffer (pH 7.5) and filtered through a 0.22 µm filter (Millipore). ThT dye was applied to 25 µL of fibril solution to the final concentration of 200 µM. Fluorescence measurements were obtained at an excitation wavelength of 442 nm and the emission spectra from 460 to 600 nm by ISS-PC1 spectrofluorometer were recorded (ISS, Champaign, IL, USA). Results were mean values ± standard error of mean (SEM) of four independent experiments.

### Time-course sedimentation assay

Pure peptides were dissolved in the purified water and placed on ice. After sonication for 1 minute, the peptide solution was centrifuged at 100,000 rpm for 3 hours at 4°C (Legend Mach 1.6R, Thermo). The supernatant of the peptide solution was obtained and quantified by UV spectrometry. 50 µM of peptide was incubated in PBS (70 mM KCl, 20 mM sodium phosphate, pH 7.0) under 37°C at different time intervals. 100 µL peptide solution was taken out each day and centrifuged at 15,000 rpm for 30 minutes to remove aggregates (Eppendorf centrifuge 5424). 75 µL of the peptide supernatant solution mixed with 25 µL 50% acetonitril were injected into HPLC to evaluate the monomeric form of the remaining TDP-43 C-terminal fragment. SEM was obtained from two independent experiments, each measured in duplicate.

### Calcein–encapsulated liposome preparations

1,2-dimyristoyl-sn-glycero-3-phosphoglycerol (DMPG) (3.3 mg), 1,2-dimyristoyl-sn-glycero-3-phosphocholine (DMPC) (5.0 mg), and cholesterol (2.5 mg) mixture were dissolves in 1∶1 chloroform/methanol and evaporated the organic solvent by nitrogen. The remaining solvent was further removed by 2 hours of lyophilization. The dried lipid film was rehydrated with 1 mL of 50 mM calcein phosphate buffer and titrated with potassium hydroxide to pH 7.0. The solution was sonicated for 1 hour and processed with seven freeze-thaw cycles with liquid nitrogen and 70°C hot plate. The suspension was extruded 19 times by Avanti Mini-Extruder through two stacked 100 nm polycarbonate membranes (Avanti Polar Lipid, Inc.) to generate homogeneous large unilamellar vesicles (LUVs). Unencapsulated calcein was removed by gel permeation chromatography (GPC) column.

### Calcein Leakage Assay

Peptides (100 µM) were preincubated for 30 minutes at 25°C and added into the liposome solution to the final lipid concentration of 0.7 mM and peptide concentration of 50 µM. Liposome was prepared in a combination of 40% DMPC, 60% DMPG and cholesterol encapsulated with self-quenching calcein. The liposome and peptides mixture were incubated in a shaking incubator at 37 °C (Eppendorf Thermomixer mixer). Fluorescence measurement was obtained by ISS-PC1 spectrofluorometer (ISS, Champaign, IL, USA) with the excitation and emission wavelength set at 490 and 520 nm. The percentage of calcein leakage is calculated as dye leakage(%)  =  (F_P_-F_L_)/(F_T_-F_L_), whereas F_P_ represent the fluorescence signal after adding the peptides, F_L_ for liposome only, and F_T_ was obtained after addition of 5% of Triton X-100.

### Cell viability assay

Mouse neuroblastoma (N2a) cells (generous gift from Dr. Yijuang Chern, IBMS, Academia Sinica) were cultured in Dulbecco's modified Eagle's medium (HyClone) supplemented with 4 mg/L L-glutamine, 1% Penicillin/Streptomycin (Invitrogen) and 10% fetal bovine serum (Invitrogen). Cells were maintained in a CO_2_ incubator with temperature set at 37°C. 100 µM of peptides were pre-incubated in phosphate buffer (70 mM KCl, 20 mM sodium phosphate, pH 7.0) at 37°C with agitation for 24 hours. 10^5^ N2a cells were then seeded in 12-well plate overnight and applied the pre-incubated TDP-43 C-terminal fragment peptides (final working concentration: 30 µM) to the cell for additional 72 hours. Cell viability was determined by AlamarBlue (AbD Serotec) assay according to manufacturer's protocol. Briefly, 10% of AlamarBlue buffer mixed with Dulbecco's modified Eagle's medium was added to each well and incubated at 37°C for 3 hours. 100 µL of aliquots were taken out from each plate and obtained the fluorescence intensity by fluorescence reader (PerkinElmer Fluorescence Spectrometer) with an excitation and emission wavelength set at 530 nm and 590 nm. Cell viability ratio was calculated as follows: cell viability  =  (sample - background)/(PBS-treated - background). The error bars indicated SEM from the means of three independent experiments.

### Time-lapse differential interference contrast imaging

10^5^ N2a cells were seeded on sterilized 35-mm glass-bottomed dish (Matsunami, Japan) for 24 hours. Meanwhile, 100 µM of peptides were pre-incubated in phosphate buffer at 37°C with agitation. The next day, pre-incubated peptides were added to the cell at a final concentration of 30 µM. Cells were incubated in a temperature-controlled system (37 °C, 5% CO_2_) to maintain its viability. Time-lapse differential interference contrast (Time-lapse DIC) images were acquired by Nikon eclipse TiE & EMCCD: Andor 888 with an exposure time of 30 ms and 10 minutes intervals for 36 hours.

### Statistical analysis

The values were shown as mean ± SEM. Statistical analysis was performed using one-way analysis of variance followed by posthoc Tukey's test.

## Results

### Pathological and *de novo* mutant (GGG308PPP) peptides from TDP-43 C-terminus formed aggregates with different morphology

**Figure 1:**
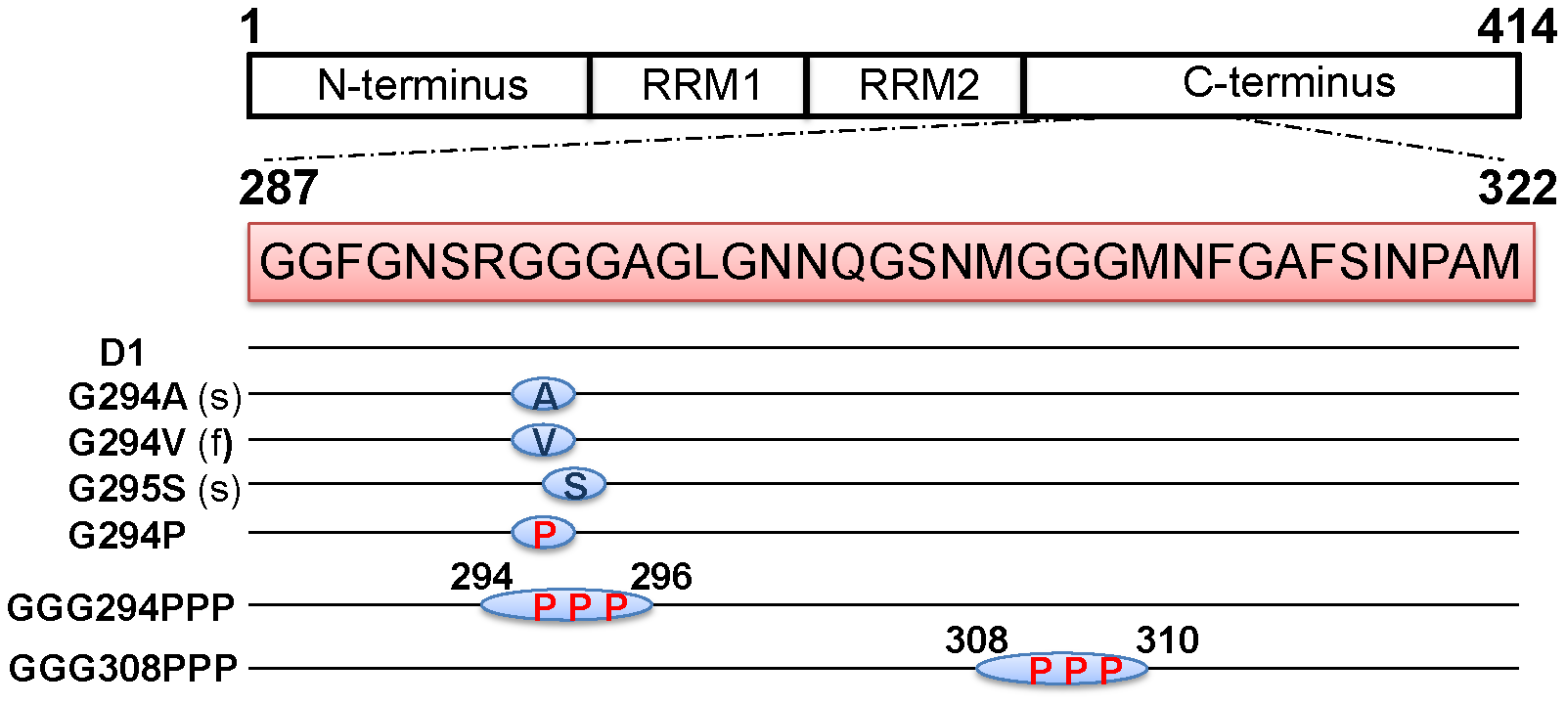
Sequence of the synthetic peptides derived from TDP-43 C-terminus with pathological and *de novo* designed mutations. The sequences of TDP-43 C-terminus peptides (residues 287–322) including D1, familial mutation (f) (G294V), two sporadic mutations (s) (G294A and G295S), as well as glycine to proline mutants (G294P, GGG294PPP, and GGG308PPP).

**Figure 2:**
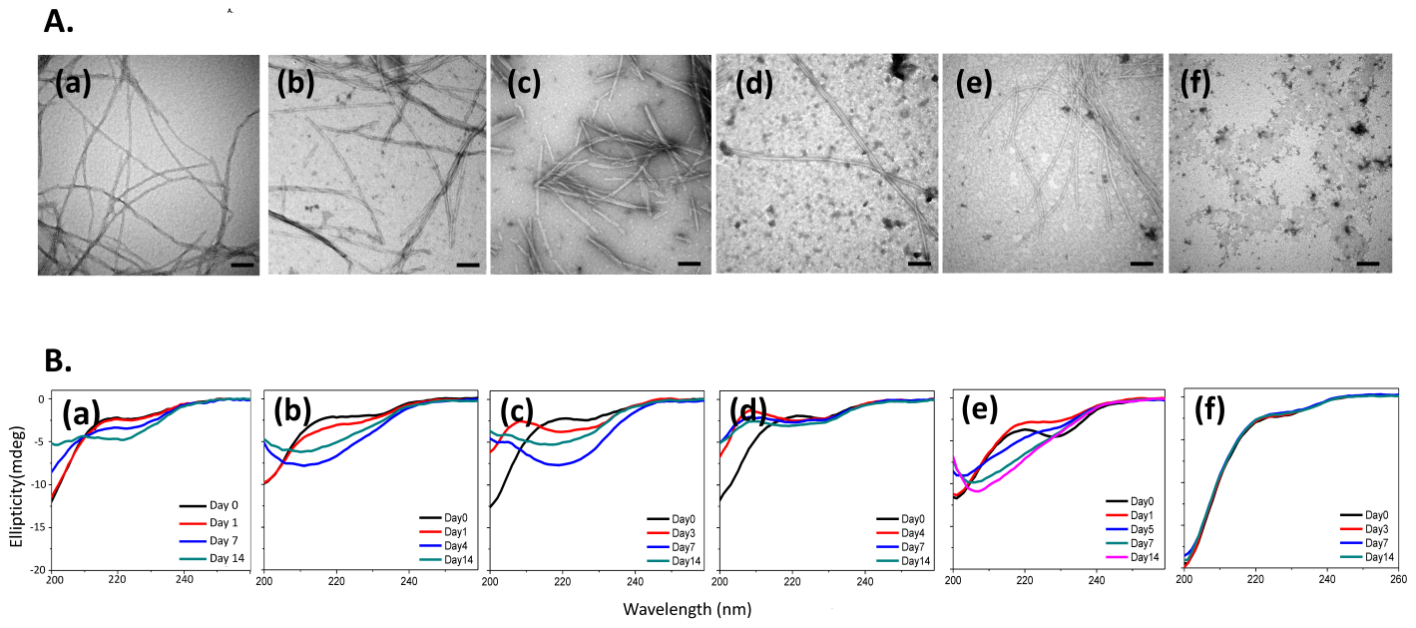
Morphological and structural characterization of different TDP-43 peptides. (A) EM of TDP C-terminus mutants (a) D1 (b) G294V, (c) G295S, (d) G294P, (e) GGG294PPP, and (f) GGG308PPP incubated in phosphate buffer at 37°C for 2 weeks. The scale bar represents 100 nm. (B) Time-course CD spectra of (a) D1 (b) G294V, (c) G295S, (d) G294P, (e) GGG294PPP, and (f) GGG308PPP.

To elucidate whether pathological mutations and the replacement of glycines by prolines are able to alter the fibrillogenesis process, we have synthesized the wild type (D1) TDP-43 C-terminus peptides and mutants containing familial (G294V), sporadic (G294A and G295S), single and triple *de novo* mutations (G294P, GGG294PPP, GGG308PPP) (Figure 1). Transmission electron microscopy (TEM) was applied to characterize the morphology of the peptide aggregates. After incubation in phosphate buffer (pH 7.0, 37°C) for 2 weeks, G294V, G295S, G294P, and GGG294PPP displayed similar fibrillar morphology [Figure 2A(b–e)] with our published glycine-rich peptides including D1 [Figure 2A(a)], G294A, and A315T [20]. While the fibrils from G294A, G294V, and G295S were all twisted, the replacement of glycine with proline (G294P, GGG294PPP) perturbed the fibrillogenesis process and induced non-twisted fibrils [Figure 2A(d–e)]. Surprisingly, only amorphous aggregates could be found in GGG308PPP [Figure 2A(f)], suggesting residues 308–310 are critical for fibril formation. We have further measured the diameter of these fibers to understand the impact of the mutations on their fibrillar morphology. The fibers from D1 and G294A displayed an average width of 11 nm while G294V and G295S showed 8 to 10 nm. Slenderer fibers, approximately 3 to 8 nm in width, were found in the two *de novo* mutants (G294P and GGG294PPP). Collectively, these data suggested pathological mutants formed typical fibrils, while the replacement of glycine with proline is able to perturb the fibrillogenesis from subtle change in fibril width to dramatic alteration in morphology.

### The glycine to proline replacement perturbed the secondary structure of its monomer/oligomer during the amyloidogenesis

While TEM provides the morphological information of the fibrils, the change in secondary structures, an important indicator to identify amyloid formation, may be monitored by circular dichroism (CD) spectroscopy. TDP-43 C-terminus peptides (D1, G294V, G295S, A315T, G294A, G294P, GGG294PPP, and GGG308PPP) were incubated in phosphate buffer and their secondary structural recorded constantly for 2 weeks. CD spectra of all peptides presented random-coiled structure at Day 0. After 14 days of incubation in phosphate buffer, D1 possessed weak β-sheet signal [Figure 2B(a)]. Similarly, all pathological mutants (G294V, G295S, A315T, and G294A) displayed notable negative ellipticity at 218 nm which indicated these mutants shift the conformation equilibrium to mostly β-sheet structure [Figure 2B(b)–(c)] [20]. The secondary structure of *de novo* mutant G294P showed mild coil-to-β transition [Figure 2B(d)] within 14 days, suggesting glycine replacement may perturb the formation of β-sheet structure. In addition, we observed a marked red-shifted negative ellipticity at 205 nm in GGG294PPP [Figure 2B(e)] at day 7, suggesting GGG294PPP may favor both polyproline type II (PPII) helix and β-sheet in its soluble state. The solvated PPII helix in protein misfolding disease has been shown to serve as an intermediate before forming β-sheet due to its extended and flexible nature [21], [22]. Therefore, we sought the replacement of triple glycines to prolines at residue 294 to 296 (GGG294PPP) retarded the β-sheet formation. On the other hand, the similar replacement at position 308 to 310 (GGG308PPP) largely obstructed the formation of β-sheet in GGG308PPP over 14 days [Figure 2B(f)]. In fact, proline is rarely found in amyloid proteins due to its rigid nature to be compatible in the β-sheet structure [23], which is in support of our data that the substitution of prolines greatly alters the monomer/oligomer structures from TDP-43 and leads to the suppression of β-sheet content.

### All fibrils were rich in β-sheet

**Figure 3:**
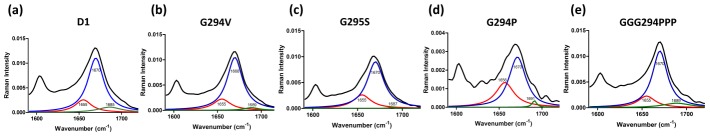
FT-Raman spectra (amide I region: 1590–1720 cm^−1^) of fibrillar aggregates from TDP-43 C-terminal fragments including (a) D1, (b) G294V, (c) G295S, (d) G294P, and (e) GGG294PPP. The black line corresponds to the original spectrum. The red, blue, and green lines represent the individual component of α-helix, β-sheet, and random coil, respectively.

Fourier transform-Raman (FT-Raman) spectrometry is another powerful tool to depict the secondary structural in peptide/protein samples. While CD may provide the ensemble of secondary structures of the analyte in the solution phase, the conformational information of the fibrils from the pellet may be obtained from FT-Raman spectroscopy. By analyzing the amide I region (1590–1720 cm^−1^) of the FT-Raman spectra, we were able to clarify the contents of α-helix, β-sheet, and random coil in each TDP-43 C-terminus mutant fibrils. After deconvolution and numerical curve fitting, all fibrils (D1, G294A, G294V, G295S, G294P, and GGG294PPP) displayed a major absorption around 1670 cm^−1^ and the β-sheet contents ranged from 67% to 78%, indicating the presence of β-sheet-rich structure in all these peptides [Figure 3(a)–(e), Table 1]. It is worth to note that the β-sheet content was not influenced by the detailed morphology of the fibers, as shown on the similar β-sheet percentage in the thicker (D1, G294A, G294V, and G295S) and slenderer fibers (G294P and GGG294PPP). Despite different β-sheet compositions of these peptides in the soluble portion as monitored by CD, their insoluble deposits showed all β-sheet-rich structures in Raman spectra. As for GGG308PPP, characterization of its aggregates by Raman spectroscopy was not practical due to its high solubility.

**Table 1:** Quantitative analysis of the secondary structure contents of the aggregates from different TDP-43 peptides determined by FT-Raman spectroscopy.

Peptides	α-helix	β-sheet	Random coil
D1	18%	76%	6%
G294V	19%	78%	3%
G295S	24%	75%	1%
G294P	30%	67%	3%
GGG294PPP	19%	76%	5%
GGG308PPP	N.D.		

N.D. Not determined.

### The glycine to proline replacement influenced the amyloid properties and aggregation rate

**Figure 4:**
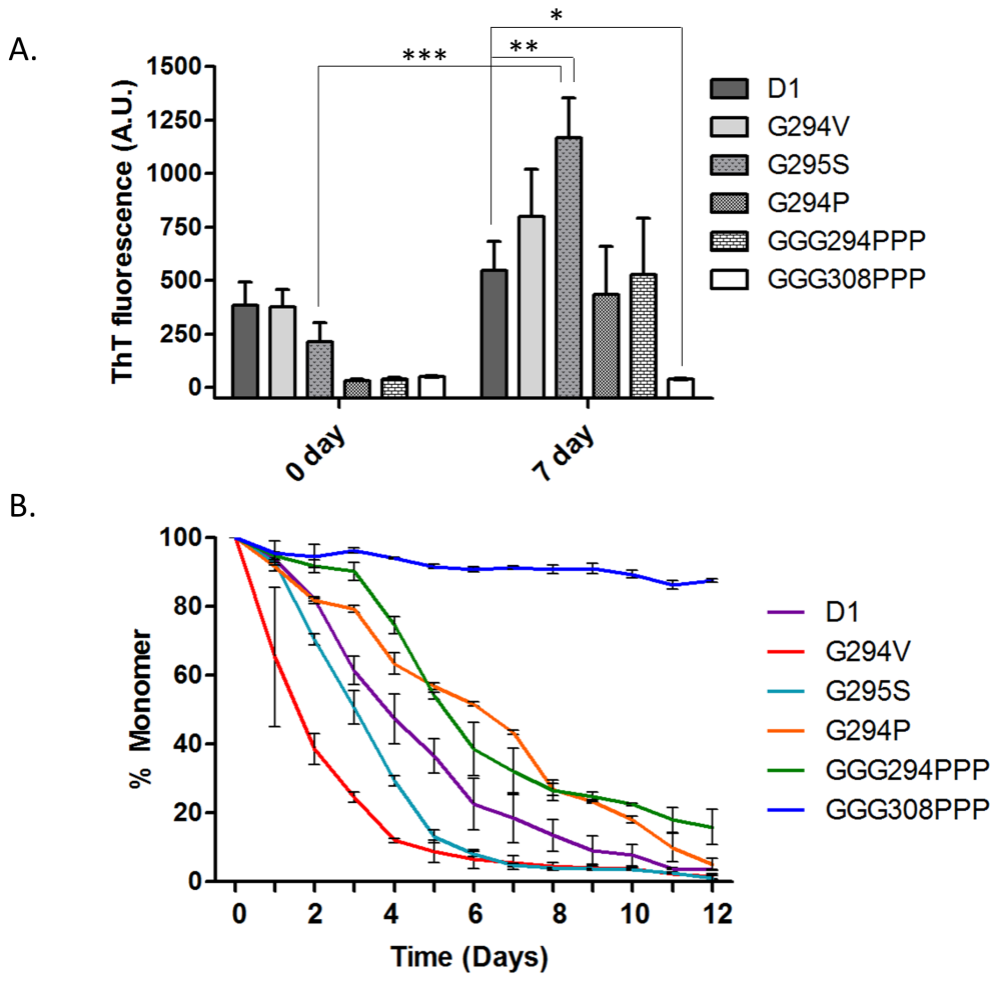
ThT and time-course sedimentation assay of TDP-43 C-terminal fragments. (A) 50 µM of each peptide was incubated in phosphate buffer at 37°C for either 0 or 7 days before the measurement of ThT fluorescence. Results were means ± SEM of four independent experiments. (*  =  p<0.05, **  =  p<0.01, ***  =  p<0.001) (B) Monitoring the remaining monomer (%) of D1 (purple), G294V (red), G295S (cyan), G294P (orange), GGG294PPP (green), and GGG308PPP (blue) by the time-course sedimentation assay.

The benzothiazole dye, thioflavin T (ThT), has commonly been applied to probe the amyloidogenic propensity of fibrils [20]. Marked increase of ThT signal can be observed in pathological mutant peptides (G294V and G295S) on the 7^th^ day of incubation in the phosphate buffer (pH 7.0, 37°C), suggesting the increase of amyloid fibers as compared to D1 (Figure 4A). In contrast, *de novo* mutants (G294P and GGG294PPP) showed reduced ThT fluorescence which may due to the presence of proline serving as a β-sheet breaker. Particularly, proline substitutions at residue 308/309/310 (GGG308PPP) exhibited extremely low ThT fluorescence intensity, suggesting triple prolines can disrupt the amyloidogenic propensity (Figure 4A).

To obtain insights in the aggregation kinetics in each TDP-43 peptide, we applied time-course sedimentation assay to monitor the content of remaining monomers during the aggregation process. Accelerated aggregation kinetics can be observed in G294A [20], G294V and G295S when comparing with D1, as less than 10% of the monomers were detected in these peptides after the 7 days (Figure 4B). However, the aggregation rates were largely retarded in the case of G294P and GGG294PPP. In particular, GGG308PPP retained mostly monomers even after 12 days of incubation (Figure 4B). From both Thioflavin T and sedimentation assay, we concluded that pathological mutants possessed strong aggregation-prone ability to convert monomer into ThT-positive oligomers/aggregates. Meanwhile, the replacement of glycine with proline in TDP-43 C-fragment, especially GGG308PPP, is able to suppress amyloidogenesis and stabilize its monomer.

### All amyloidogenic peptides exhibited membrane disruption ability

**Figure 5:**
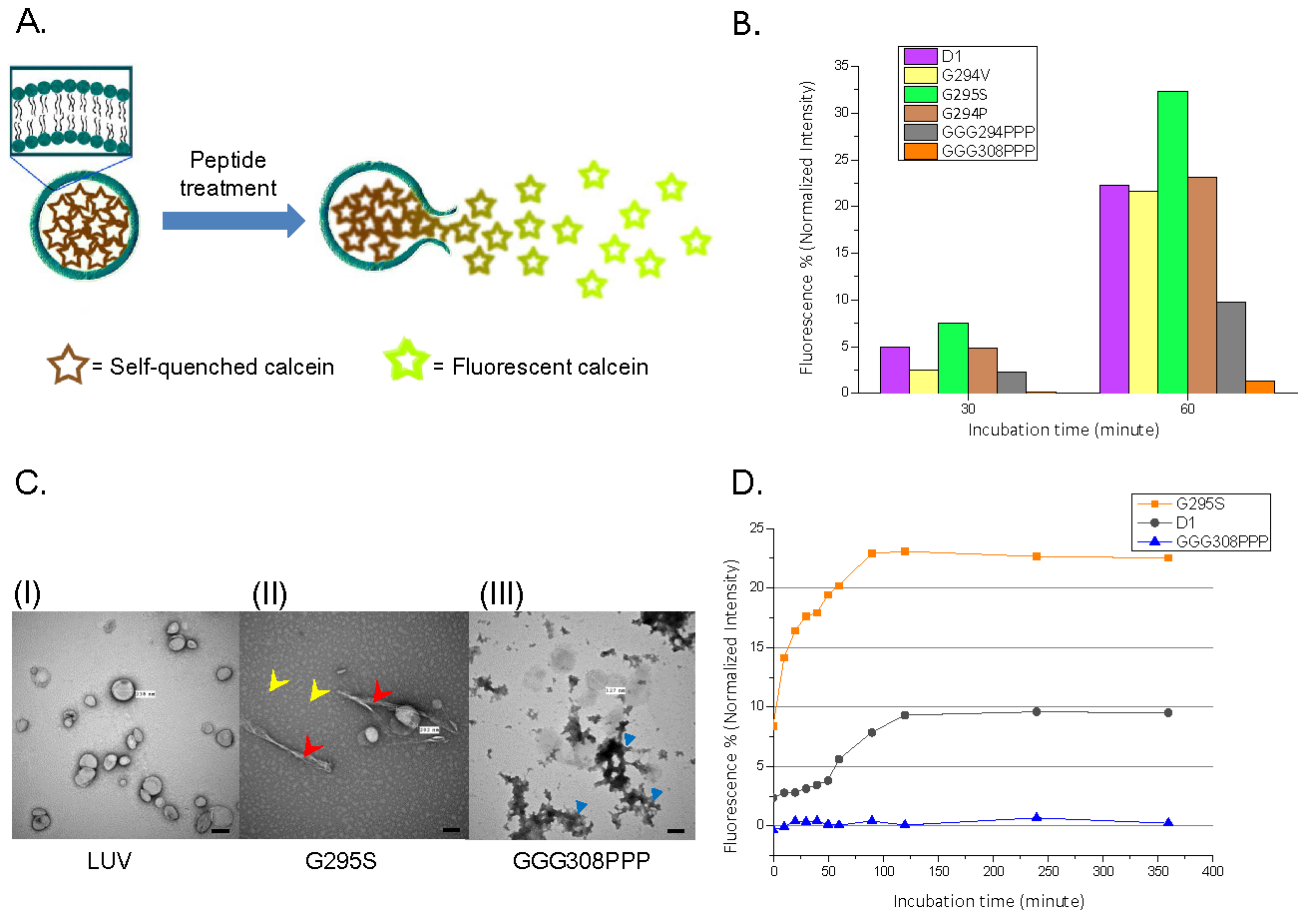
The induce of calcein leakage and liposome disruption with amyloidogenic peptides. (A) Upon the disruption of the membrane, the self-quenched calcein is released from the liposome and generate strong fluorescence signal. (B) The induced calcein leakage (%) was monitored in the presence of different peptides after 30 or 60 minutes incubation. (C) Freshly extruded large unilamellar vesicles (LUV) were identified either alone (left) or in the presence of G295S (middle) or GGG308PPP (right). The scale bars represent 100 nm. (D) Time-course of calcein fluorescence enhancement in the presence of D1, G295S, and GGG308PPP (50 µM).

Recent researches have indicated TDP-43 C-terminus (277–414) as a prion-like domain which played a critical role in TDP-43 pathogenesis [24]–[26]. To obtain further insights in whether TDP-43 C-terminus mutant peptides may possess prion property as PrP^sc^ (scrapie isoform of prion protein) in membrane disruption, we applied calcein leakage assay to monitor the membrane permeability in the presence and absence of different peptides. As shown in Figure 5A, the peptides that cause the disruption of biomimetic membrane in liposome will lead to calcein efflux and increase its fluorescence. Large unilamellar vesicles (LUV) were observed under TEM (Figure 5C, left panel). Different peptides (D1, G295S, G294P, G294V, GGG294PPP, and GGG308PPP) were co-incubated with liposomes followed by fluorescence measurement (Ex/Em  =  490/520 nm). Within these peptides, stronger liposome leakage (∼8%) is found in the presence of G295S; moderate (∼4%) in D1, G294V, G294P, and GGG294PPP; trace in GGG308PPP at 30 minutes. Similar trend can be observed at 60 minutes of incubation while the strongest liposome disruption reached to 30% in G295S; 10∼20% in D1, G294V, G294P, and GGG294PPP; ∼2% in GGG308PPP (Figure 5B). Peptides with the strongest (G295S) and weakest (GGG308PPP) membrane disruption ability have been incubated with the liposome solution and characterized their morphological change by TEM. G295S demonstrated a population of oligomers (Figure 5C, middle panel, yellow arrow) and fibrils (Figure 5C, middle panel, red arrow) attaching to biomimetic membranes, whereas only amorphous aggregates were found in GGG308PPP (Figure 5C, right panel, blue triangle). In addition, a time-trace fluorescence leakage assay was conducted to monitor the induction of membrane disruption by TDP-43 peptides. Fluorescence increase in G295S and D1 reached their plateau at ∼100 minutes while the vesicle remained its integrity in the presence of GGG308PPP over time. Meanwhile, G295S displayed shorter half-time of membrane disruption (40 minutes) when comparing with D1 (70 minutes). (Figure 5D). Our data showed that pathological mutant (G295S) exhibited a stronger ability to destabilize biomimetic membrane than wild-type peptide (D1), whereas triple proline mutant (GGG308PPP) could hardly disrupt the liposome.

### The pathological role of TDP-43 C-terminus mutants

**Figure 6:**
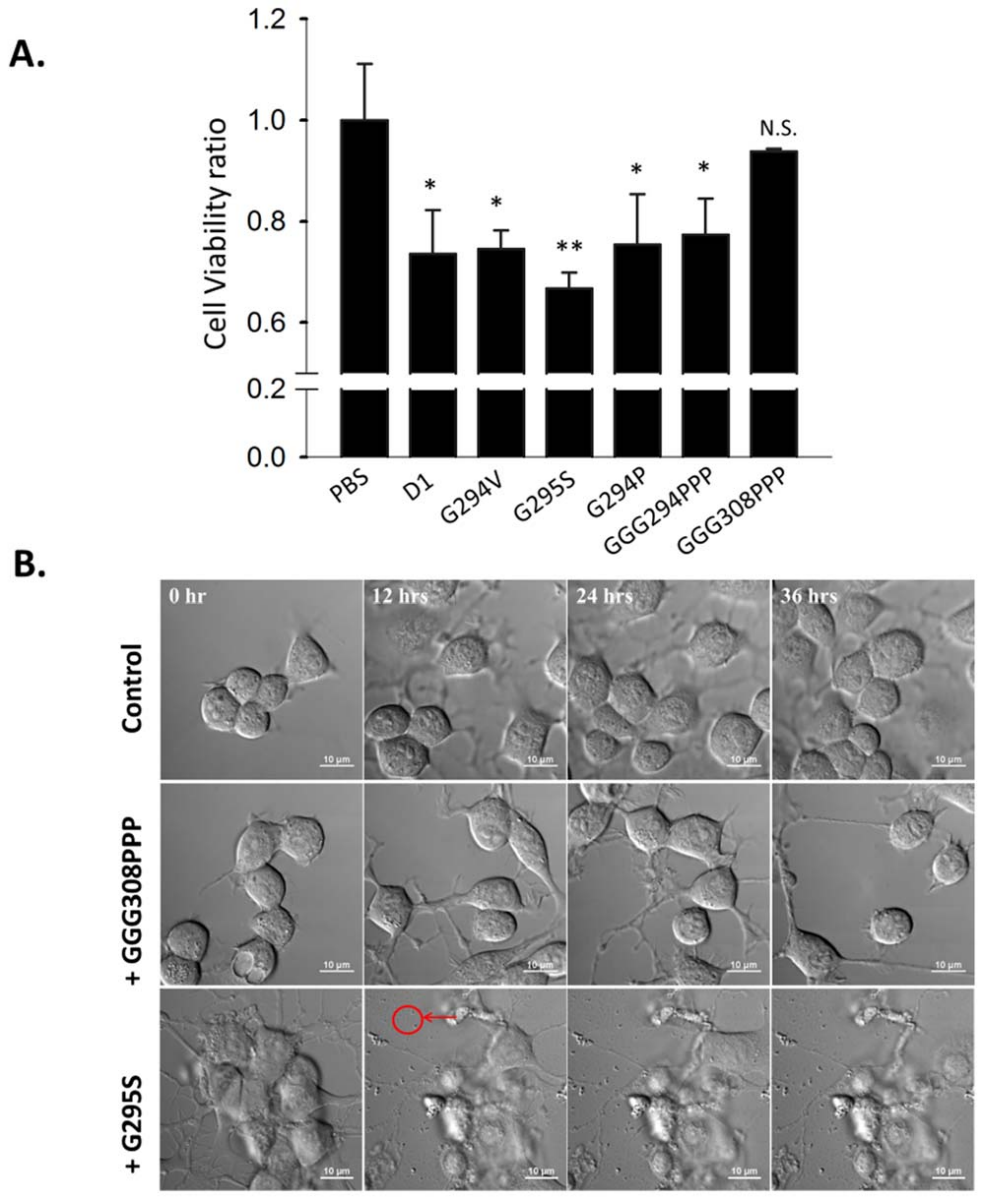
TDP-43 pathological and glycine to proline mutant peptides exhibit different neurotoxicity. (A) The comparison of cell viability in the presence of D1, G294V, G295S, GGG294PPP, and GGG308PPP for 72 hours determined by AlamarBlue assay. Results were means ± SEM of three independent experiments (*  =  p<0.05, **  =  p<0.01, N.S.  =  not significant). (B) Time-lapse DIC microscopy of N2a cell after the addition of PBS, GGG308PPP, or G295S for 36 hours.

We have previously demonstrated TDP-43 C-terminus fragment (287–322) exerted cytotoxicity toward N2a cells [20]. To further delineate the possible neurotoxic effect of TDP-43 mutants (G294V, G295S) and the proline-substituted peptides (G294P, GGG294PPP, GGG308PPP), we have added these peptides to N2a cells and examined the cell viability after 72 hours. Despite the distinct structure and amyloidogenicity among D1, G294V, G294P, and GGG294PPP, these peptides significantly hampered the survival of N2a cell by 25% (p<0.05, Figure 6A) while G295S decreased ∼35% cell viability after 72 hours (p<0.01, Figure 6A). Conversely, GGG308PPP displayed no significant reduction of cell survival compared with PBS treatment, suggesting the proline substituent at residue 308–310 prohibited the neurotoxicity from TDP-43 C-terminal peptides.

To further obtain insights in how mutant peptides affect cell morphology, we applied time-lapse differential interference contrast (DIC) microscopy to visualize the morphological changes of N2A after the addition of peptides (G295S and GGG308PPP) for 36 hours. As expected, in the presence of GGG308PPP, which formed amorphous aggregates and exhibited lowest neurotoxicity, no obvious morphological alterations were observed at the 12^th^, 24^th^, and 36^th^ hour (Figure 6B). In contrast, small aggregates/fibers (Figure 6B, red circle) along with the cellular shrinkage and cell death could be found from the 12^th^ to 36^th^ hour in the G295S-treated cells, correlating with our cell viability results. Hence, our cell viability, Thioflavin T assay, along with time-lapse DIC microscopy result indicated that the structural conversion from soluble monomer to amyloid fibrils in G295S and G294V may be the causative factor to jeopardize the cell morphology and viability. Glycine to proline replacement in the critical sites in TDP-43 (GGG308PPP) suppressed the amyloidogenic process which further reduced neurotoxicity.

## Discussion

Our results provided both the biophysical and biochemical information of TDP-43 C-terminus mutant peptides. We have previously reported that a specific fragment (residue 287–322) from TDP-43 C-terminus, D1, is able to form twisted fibers and β-amyloid structure through the analysis of TEM and CD [20]. In this study, the impact of pathological (G294V, G295S) and *de novo* mutations (G294P, GGG294PPP, GGG308PPP) on the amyloid properties and cytotoxicity of TDP-43 glycine-rich peptides was characterized. We also explored the “prion-like” propensity in TDP-43 pathological mutants, G294V and G295S, which exerted strong ability to disrupt liposome integrity and cause neurotoxicity.

### The amyloid and prion-like properties of TDP-43 peptide fragments

The TDP-43 C-terminal domain (residue 277–414) has been proposed as a prion-like domain, exhibiting prionogenic property and sharing 24.2% sequence homology with the prion domain in Sup35 [24], [26]. However, the intrinsic property of TDP-43 has always been argued due to its negativity to Congo staining [13]. Recently, accumulating evidences showed TDP-43 positive inclusions co-localized with Thioflavin S in the brain tissue of FTLD-TDP (Frontotemporal Lobar Degeneration with TDP-43 proteinopathy) and ALS cases, which provided the histological evidence in the amyloid property of TDP-43 [27], [28]. Synthesized peptides derived from the specific prion-like domain of TDP-43 also displayed the ability to form amyloid fibers, supporting its prionogenic property *in vitro* [20], [29]. The prion-like characteristics have been previously disclosed in other amyloid peptides [such as Amyloid-β (Aβ), PrP^sc^, and α-synuclein] to associate with cell membrane and forming pore-like structures, which further leads to the disruption of ionic homeostasis through membrane leakage, increases ROS and calcium influx, and induces pathophysiological degeneration [30]–[34]. The intrinsic nature of TDP-43 C-terminus has been characterized to possess prion-like property, which further hint on the possible TDP-43 proteinopathy in early pathogenesis[35]. While numerous pathological mutations have been found in ALS patients, the pathological impact in these mutations remained unsolved.

Our study is the first to report the two TDP-43 C-terminus mutant peptides (G294V and G295S) displayed enhanced amyloidogenic and prion-like characteristics, which may contribute a toxic gain-of-function in TDP-43 proteinopathy. These two mutants underwent significant coil-to-beta conformational transition and exerted strong biomimetic liposome destabilization ability as well as neurotoxicity (Figure 2 and 5, Table S1 in File S1). We believed that the oligomers/fibrils from these mutants may contribute to membrane disruption and induce cell death though the detailed mechanism in how they enter N2a cell and affect biological system required further investigation. Moreover, the pathological mutations conducted in this study were located in the glycine-rich domain (residues 274–314), whereas this region has been reported structurally similar to heterogeneous nuclear ribonuclear proteins (hnRNPs) [36], [37]. The interaction between TDP-43 and hnRNPA2 is important in maintaining cellular homeostasis while alterations in TDP-43-recruited hnRNP complex formation may perturb downstream RNA splicing regulation and protein-protein interactions [38], [39]. Future experiments will be applied to dissect how structural aberrations and prion-like properties in TDP C-terminus mutants (G294V and G295S) influence the interaction with their downstream cellular binding partners in TDP-43 proteinopathy.

### The replacement of glycines by prolines altered the aggregation propensity

Despite the morphological similarity among TDP-43 C-terminus mutants (GGG308PPP), their structural conversion and biochemical property greatly differs. From ThT fluorescence and HPLC sedimentation assay, we discovered that proline substituents (G294P, GGG294PPP, and GGG308PPP) showed profound decreased aggregation ability which indicated that proline may perturb the intermolecular association during amyloid formation. We thus suggested that proline replacement may reduce the overall flexibility of the peptide. Due to the cyclic side chain in the proline, its φ backbone dihedral angle was restricted from −90° to −60°. Increased conformational rigidity is incompatible in β-sheet structure and may hence influence the amyloidogenesis process [40], [41]. The addition of proline residues have also been shown to block β-sheet propensity and prevent fibrillogenesis in the case of Aβ, polyglutamine, and human islet amyloid polypeptide (hIAPP) [22], [42]–[45]. Furthermore, proline replacement has shown to efficiently inhibit amyloidogenesis and increase solubility, which may be served as a possible strategy to suppress the off-pathway aggregation in amyloid polypeptides [46]–[48].

In our work, proline substituents (G294P, GGG294PPP, and GGG308PPP) showed profound effects in decreasing aggregative and disrupted the non-native β-dominant structure. It is noteworthy that proline substitution at residue 308/309/310 in TDP-43 (GGG308PPP) may lead to dramatic morphological change when comparing to other proline substituents (G294P, GGG294PPP) and wild type (D1). Subsequent and functional analysis also displayed strong ability to stabilize solubility, amyloid formation, membrane destabilization, and cytotoxicity in TDP-43 C-terminus mutants, indicating the crucial role of glycines (position 308–310) in amyloid fibrillogenesis and neuronal death.

The impact of protein sequence in protein folding has long been discussed whereas certain amino acids are critical for inducing misfolded structure. The replacement of these specific amino acids to proline allows the polypeptide to populate an alternative conformation, which may due to the increase of unfolded-to-misfolded energy barrier [47]. This is in support of our data as we speculate that proline replacement in certain sites of TDP-43 may have disrupted the disordered folding pathway of mutant TDP-43 to an alternative folding that is non-toxic to the cellular system. Interestingly, we have recently co-incubated the GGG308PPP with G295S and observed the morphological change of both peptides under EM. The presence of GGG308PPP suppressed the fibril formation of G295S and transformed it into amorphous aggregates (Figure S1 in File S1). The detailed mechanism in whether GGG308PPP binds to G295S and how the fibrillation process is perturbed is still under investigation.

Conclusively, we have characterized in detail the structural and biological properties of ALS-linked mutations in TDP-43 proteinopathy, which may provide insight in the disease pathogenesis. Our approach using glycine to proline replacement to perturb amyloidosis may shed light on establishing the linkage between structural aberrations and pathophysiological mechanism for the future therapeutic development.

## Supporting Information

File S1 **Supporting Information File. Table S1. Summary of the biophysical/biological characteristics of the TDP-43 C-terminal mutant and proline substituents fragments. Figure. S1 Co-incubation of GGG308PPP with pathological mutant G295S resulted in massive amorphous aggregate formation.** Electron micrographs of (A) G295S only or (B) co-incubated with GGG308PPP in a 4∶1 molar ratio at 37°C. Fibril solutions were negatively stained with 2% uranyl acetate and observed by Hitachi H-7000 electron microscope. The scale bars represent 100 nm.
(DOC)
