# Supplementary material for: The Influence of Pathological Mutations and Proline Substitutions in TDP-43 Glycine-Rich Peptides on Its Amyloid Properties and Cellular Toxicity
Source: PLoS One. 2014 Aug 4;9(8):e103644. doi: 10.1371/journal.pone.0103644 (PMC4121164; doi:10.1371/journal.pone.0103644)

**Supplementary information**

The influence of pathological mutations and proline substitutions in TDP-43 glycine-rich peptides on its amyloid properties and cellular toxicity

**Chia-Sui Sun^1,2,3,4^, Cindy Yu-Hsiang Wang^2,5^, Bryan Po-Wen Chen^2^*,* Ruei-Yu He*^2^*, Chih-Hsien Wang*^6^*,** **Gerard Chun-Hao Liu^2,5^, Wenlung Chen*^6^*, Yijuang Chern^3^, Joseph Jen-Tse Huang^2*^**

From the ^1^Taiwan International Graduate Program in Molecular Medicine, National Yang-Ming University and Academia Sinica, Taipei, Taiwan, ^2^Institute of Chemistry and ^3^Institute of Biomedical Sciences, Academia Sinica, Taipei, Taiwan, ^4^Institute of Biochemistry and Molecular Biology, National Yang-Ming University, Taipei, Taiwan, ^5^Department of Chemistry, National Taiwan University, Taipei, Taiwan, **^6^**Department of Applied Chemistry, National Chiayi University, Chiayi, Taiwan,

To whom correspondence should be addressed: Joseph Jen-Tse Huang, Institute of Chemistry, Academia Sinica: No.128, Sec. 2, Academia Road, Nankang, Taipei 11529, Taiwan. Tel: 886-2-27898652; Fax: 886-2-27831237; E-Mail: [jthuang@gate.sinica.edu.tw](mailto:jthuang@gate.sinica.edu.tw)

**Table S1.**

**Summary of the biophysical/biological characteristics of the TDP-43 C-terminal mutant and proline substituents fragments.**

| **Sequence 287-322** | **D1** | **G294V (f)** | **G295S (s)** | **G294P** | **GGG294PPP** | **GGG308PPP** |
| --- | --- | --- | --- | --- | --- | --- |
| TEM | Fibril [19] | Fibril | Fibril | Fibril | Fibril | Amorphous aggregates |
| CD spectroscopy | Weak β-sheet [19] | β-sheet | β-sheet | Weak β-sheet | PPII-β strand | Random coil |
| FT-Raman  (β-sheet content) | 78% [19] | 78% | 75% | 67% | 76% | N.D. |
| ThT assay | Weak positive [19] | Positive | Positive | Weak positive | Weak positive | Negative |
| Time course sedimentation assay | Medium | Fast | Fast | Slow | Slow | Very slow |
| Cell viability assay | Toxic | Toxic | Toxic | Toxic | Toxic | Non-toxic |

N.D. Not determined

**Fig. S1**

**Co-incubation of GGG308PPP with pathological mutant G295S resulted in massive amorphous aggregate formation.** Electron micrographs of (A) G295S only or (B) co-incubated with GGG308PPP in a 4:1 molar ratio at 37℃. Fibril solutions were negatively stained with 2% uranyl acetate and observed by Hitachi H-7000 electron microscope. The scale bars represent 100 nm.


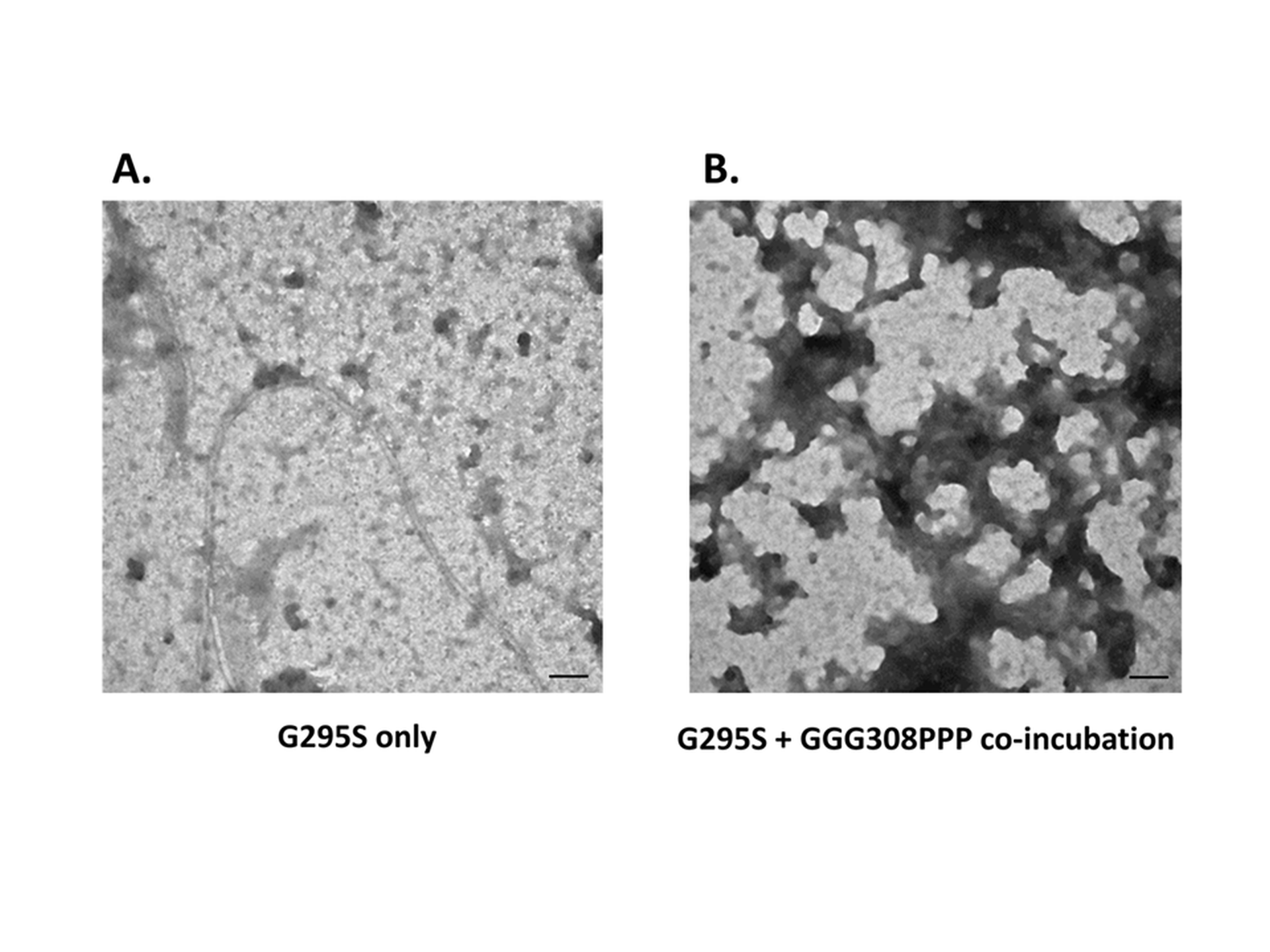

Supplement: File S1 — Supporting Information File. Table S1. Summary of the biophysical/biological characteristics of the TDP-43 C-terminal mutant and proline substituents fragments. Figure. S1 Co-incubation of GGG308PPP with pathological mutant G295S resulted in massive amorphous aggregate formation. Electron micrographs of (A) G295S only or (B) co-incubated with GGG308PPP in a 4∶1 molar ratio at 37°C. Fibril solutions were negatively stained with 2% uranyl acetate and observed by Hitachi H-7000 electron microscope. The scale bars represent 100 nm. (DOC) [file pone.0103644.s001.doc]
